# Rheumatoid arthritis and coronary atherosclerosis: a two-sample Mendelian randomization study

**DOI:** 10.3389/fcvm.2023.1033644

**Published:** 2023-04-28

**Authors:** Qiong Nie, Qiang Luo, Wei Yan, Tongtong Zhang, Han Wang, Jing Wu

**Affiliations:** ^1^ Department of Geriatrics, The Affiliated Hospital of Southwest Jiaotong University, The Third People's Hospital of Chengdu, Chengdu, China; ^2^ Department of Cardiology, The Affiliated Hospital of Southwest Jiaotong University, The Third People's Hospital of Chengdu, Chengdu, China; ^3^ Department of General Surgery, The Center of Gastrointestinal and Minimally Invasive Surgery, The Affiliated Hospital of Southwest Jiaotong University, The Third People's Hospital of Chengdu, Chengdu, China

**Keywords:** rheumatoid arthritis, coronary atherosclerosis, mendelian randomization, genome-wide-association studies, causation

## Abstract

**Objectives:**

The relationship between rheumatoid arthritis (RA) and coronary atherosclerosis is widely concerned, but observational studies have not clarified causality. We performed two-sample Mendelian randomization (MR) study to assess the causal association between RA and coronary atherosclerosis.

**Methods:**

we mainly conducted MR analysis using the inverse variance weighted (IVW) approach. Weighted median, MR-Egger regression and maximum likelihood were conducted as sensitivity analyses for supplementary analysis. Multivariate MR also were performed to validate the results of two-sample MR. Furthermore, we performed the MR-Egger intercept, MR-PRESSO, Cochran's Q test, and “Leave-one-out” to assess the levels of pleiotropy and heterogeneity.

**Results:**

IVW result showed a positive link between genetic predisposition to RA and increased relative risk of coronary atherosclerosis (OR: 1.0021, 95%CI 1.0011-1.0031, P < 0.05). The result was confirmed by the weighted median method (OR: 1.0028, 95%CI 1.0014-1.0042, P < 0.05), MR-Egger regression (OR: 1.0031, 95%CI 1.0012-1.0049, P < 0.05) and maximum likelihood (OR: 1.0021, 95%CI 1.0011-1.0030, P < 0.05). Multivariate MR also reached a consistent conclusion. In addition, MR-Egger intercept (P = 0.20) and MR-PRESSO (P = 0.06) did not provide evidence of horizontal pleiotropy. Meanwhile, the results of Cochran's Q test (P = 0.05) and “Leave-one-out” failed to detect significant heterogeneity.

**Conclusion:**

The result of the two-sample MR analysis found genetic evidence to support the positive causal association between RA and coronary atherosclerosis, suggesting that active intervention for RA may reduce the incidence of coronary atherosclerosis.

## 1. Introduction

Rheumatoid arthritis (RA) is an immune disease mediated by autoimmunity, characterized by joint and extra-articular damage. Globally, the age-standardized prevalence rate is 224.25 (95%UI: 204.94–245.99) in 2019. Regarding the incidence, the age-standardized incidence rate is 13 per 100,000 people (95%UI: 11.83 to 14.27) (1). The peak incidence of RA is between the age of 30 to 50. It is more common in females.

Cardiovascular disease is one of the leading causes of death in RA patients (2). Several observational studies have shown that RA patients increased the risk for mortality and morbidity of cardiovascular disease by 50% and 60%, respectively, compared to the control population (3, 4). Coronary atherosclerosis is a very vital pathological basis in the development and progression of cardiovascular disease. In a case-control study of 150 RA patients, the coronary plaque burden of RA patients was higher than non-RA patients (71% vs. 45%, *P* < 0.05) (5). Another case-control study also found that coronary artery calcification was more frequent and severe in RA patients with a longer course of the disease (OR: 3.42, 95%CI 1.55–7.53, *P* < 0.05), which was independently associated with cardiovascular risk factors (6). Lately, a meta-analysis with 788 RA patients proved that RA patients had higher coronary calcium scores (RR: 48.25, 95%CI 26.97–69.53, *P* < 0.05) (7). In addition, occult coronary plaque may be more susceptible to rupture and damage in RA patients than in the control population (8). However, most of these previous studies were clinical observational studies, which could not avoid reverse causality and confounding factors in clinical studies (9). Therefore, the cause of RA and coronary atherosclerosis is uncertain.

Mendelian randomization (MR) is an analysis that utilizes genetic variants as an instrumental variable (IV) to explore the causal association of exposure with the outcome (10). Compared to randomized controlled trials, MR has the advantage of being cost-saving, time-saving, and high feasibility. In genetic correlations, the direction of causality is specific and not susceptible to confounding such as social and psychological factors. Therefore, MR can minimize unmeasured confusion and avoid reverse causality bias in observational studies (11), which occupies a unique position in the hierarchy of clinical evidence. Thus, we performed a two-sample MR analysis to investigate the causal relationship between RA and coronary atherosclerosis susceptibility in the present study.

## 2. Materials and methods

### 2.1. Study design

The study analyzed the causal effect of RA on coronary atherosclerosis using a two-sample MR analysis. RA as an exposure factor and coronary atherosclerosis as an outcome factor in our analysis. We followed the Strengthening the Reporting of Observational Studies in Epidemiology (STROBE)- MR Statement (12) (Supplementary Table S1).

### 2.2. Data source

We selected genetic variants associated with RA from a large meta-analysis of genome-wide association studies (GWAS), including 58,284 samples (14,361 RA cases and 42,923 controls) and 9,700,598 SNPs (13). The diagnosis of RA was defined according to the 1987 RA diagnostic criteria of the American College of Rheumatology (14). Then Genetic data for coronary atherosclerosis were obtained from the UK Biobank, a large-scale prospective cohort study recruiting more than 500,000 participants from the UK general population between 2006 and 2010 (15). Summary statistics for coronary atherosclerosis included 361,194 samples (14,334 cases and 346,860 controls) and 13,586,589 SNPs. To minimize the bias caused by population stratification, we included pooled data from the population of European ancestry. A detailed description of the data sources had been summarized in Table 1.

**Table 1:** Description of data used for the two phenotypes.

Trait	GWAS ID	Sample size	ncase	ncontrol	Number of SNPs	Population
Rheumatoid arthritis	ebi-a-GCST002318	58,284	14,361	42,923	9,700,598	Europeans
Coronary atherosclerosis	ukb-d-I9_CORATHER	361,194	14,334	346,860	13,586,589	Europeans

GWAS, genome-wide association studies; SNP, single nucleotide polymorphisms.

### 2.3. Instrumental variables selection

**Figure 1:**
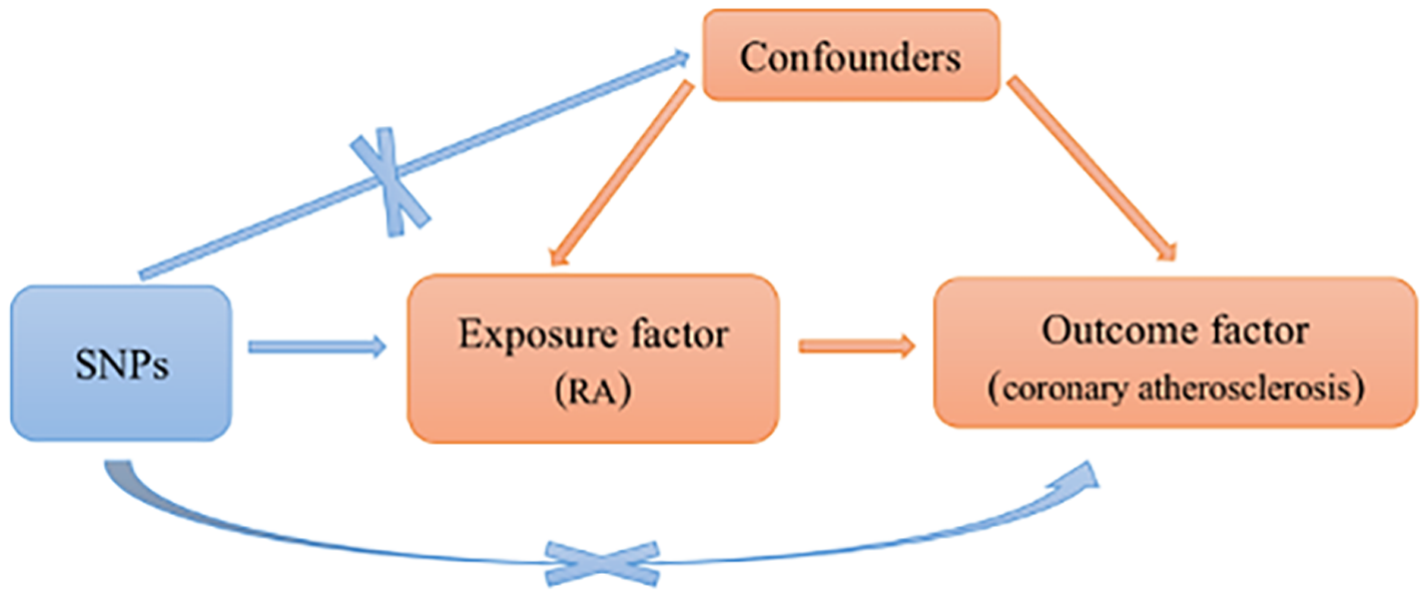
Schematic representation of an MR analysis. Three basic assumptions of MR analysis. MR, Mendelian randomization; RA, rheumatoid arthritis; SNPs, single nucleotide polymorphisms.

Single nucleotide polymorphisms (SNPs) were included based on the following assumptions: SNPs were significantly associated with RA. SNPs were not related to coronary atherosclerosis. Only RA can affect coronary atherosclerosis. Those SNPs with incompatible alleles and those with palindromic allele frequencies of no more than 0.5 were excluded from the analysis (shown in Figure 1).

We extracted SNPs as IVs associated with RA with genome-wide significant levels (*P* < 5 × 10^−8^). Then we set the threshold for linkage disequilibrium at *R*^2^ < 0.001 to exclude the effect of linkage disequilibrium. Furthermore, we investigated whether the included SNPs were associated with confounders using Catalog and Phenoscanner, and if not, the SNP were included. Finally, the F-statistic was conducted to assess the strength of IVs. When F-value was less than 10, indicating a severe bias in the causal estimation (16).

### 2.4. Ethnic statement

Our pooled data were derived from published studies approved by the institutional review board in the respective studies. Therefore, ethical approval was not required for this article.

### 2.5. MR analysis

#### 2.5.1. Effect size estimates

A two-sample MR analysis was performed mainly using the inverse variant weighted (IVW) method. IVW is the standard method for pooling data from MR, which assumes each SNP is a valid IV (17). If there was no heterogeneity, the IVW fixed-effect model was preferred. Otherwise, the random-effect model. Furthermore, we performed a series of sensitivity analyses. More specifically, MR-Egger regression was used to assess the robustness of the IVW result. However, the estimates of MR-Egger regression exhibited low precision. Therefore, we also used weighted median method to explore the reliability of the result (even though up to 50% of genetic variants were considered invalid instruments). Compared with the IVW method, maximum likelihood fully takes into account the uncertainty of SNP-exposure association, which is ignored in the simple weighting of IVW (18).

#### 2.5.2. Assessment of horizontal pleiotropy and heterogeneity

To make our results more reliable, we performed the MR-Egger intercept test and MR-PRESSO to explore directional horizontal pleiotropy. When the *P*-value of MR-Egger intercept was below 0.05, indicating the existence of horizontal pleiotropy. The MR-PRESSO global test was used to detect the existence of horizontal pleiotropy (19). Meanwhile, we provided funnel plots of the effect estimation for each SNP for visual inspection of pleiotropy. Cochran's Q test was also performed to quantitively assess the heterogeneity of causal effect sizes between the genetic instruments. In addition, “Leave-one-out” analysis investigated the effect size of the remaining SNPs on the results by excluding individual SNPs.

The results were presented as odds ratios (OR) and 95% confidence interval (CI). Statistical analyses were conducted using the package of TwoSampleMR in R software.

## 3. Results

### 3.1. SNP selection

In this study, 54 SNPs were taken as IVs. The F statistics value for every IV was much larger than 10 (the range was 25.9393 to 722.9848), suggesting the possibility of weak IVs being present was low. The result showed that the included IVs could explain 5.79% of the genetic variation.

Characteristics of SNPs included were provided in Supplementary Table S2.

### 3.2. MR statistical results

**Figure 2:**
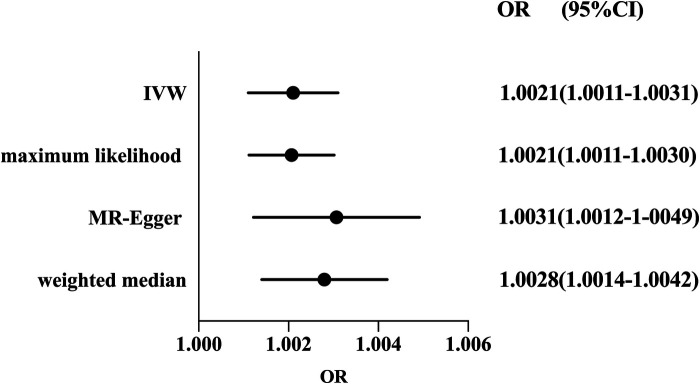
Forest plot of the effect of RA on coronary atherosclerosis risk. Forest plots of main and all sensitivity analyses. Estimates were obtained using a fixed-effects model. The black dots represent the OR value obtained by each method and the solid line represents the 95% CI. IVW, inverse variance weighted; MR, Mendelian randomization; OR, odds ratio; CI, confidence interval.

Considering the absence of heterogeneity, we used a fixed-effect model for IVW. The result of MR suggested that RA was a risk factor for susceptibility to coronary atherosclerosis (OR: 1.0021, 95%CI 1.0011–1.0031, *P* < 0.05). The causal association was consistent with sensitivity analyses using the weighted median method (OR: 1.0028, 95%CI 1.0014–1.0042, *P* < 0.05), MR-Egger regression (OR: 1.0031, 95%CI 1.0012–1.0049, *P* < 0.05) and maximum likelihood (OR: 1.0021, 95%CI 1.0011–1.0030, *P* < 0.05) (shown in Figure 2).

### 3.3. Horizontal pleiotropy and heterogeneity

**Figure 3:**
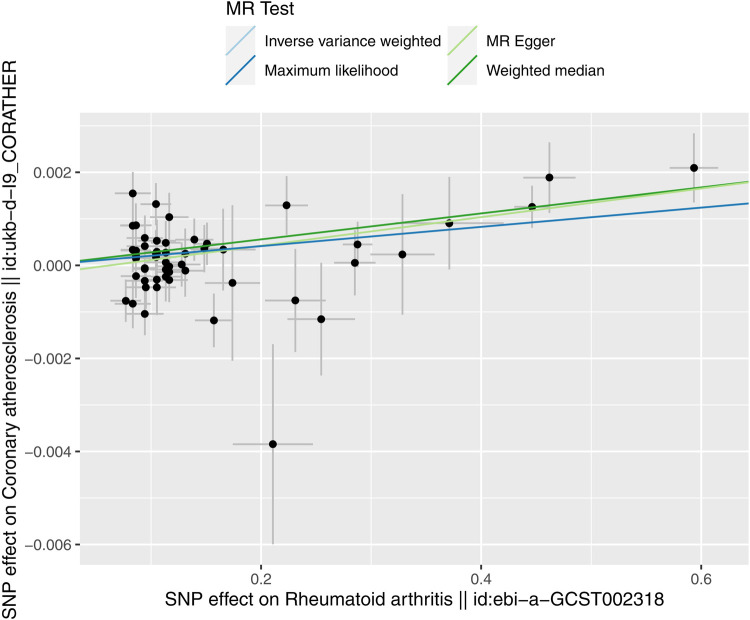
Scatter plot for RA on coronary atherosclerosis. The slope of each line corresponds to the MR effect estimated by each method.

**Figure 4:**
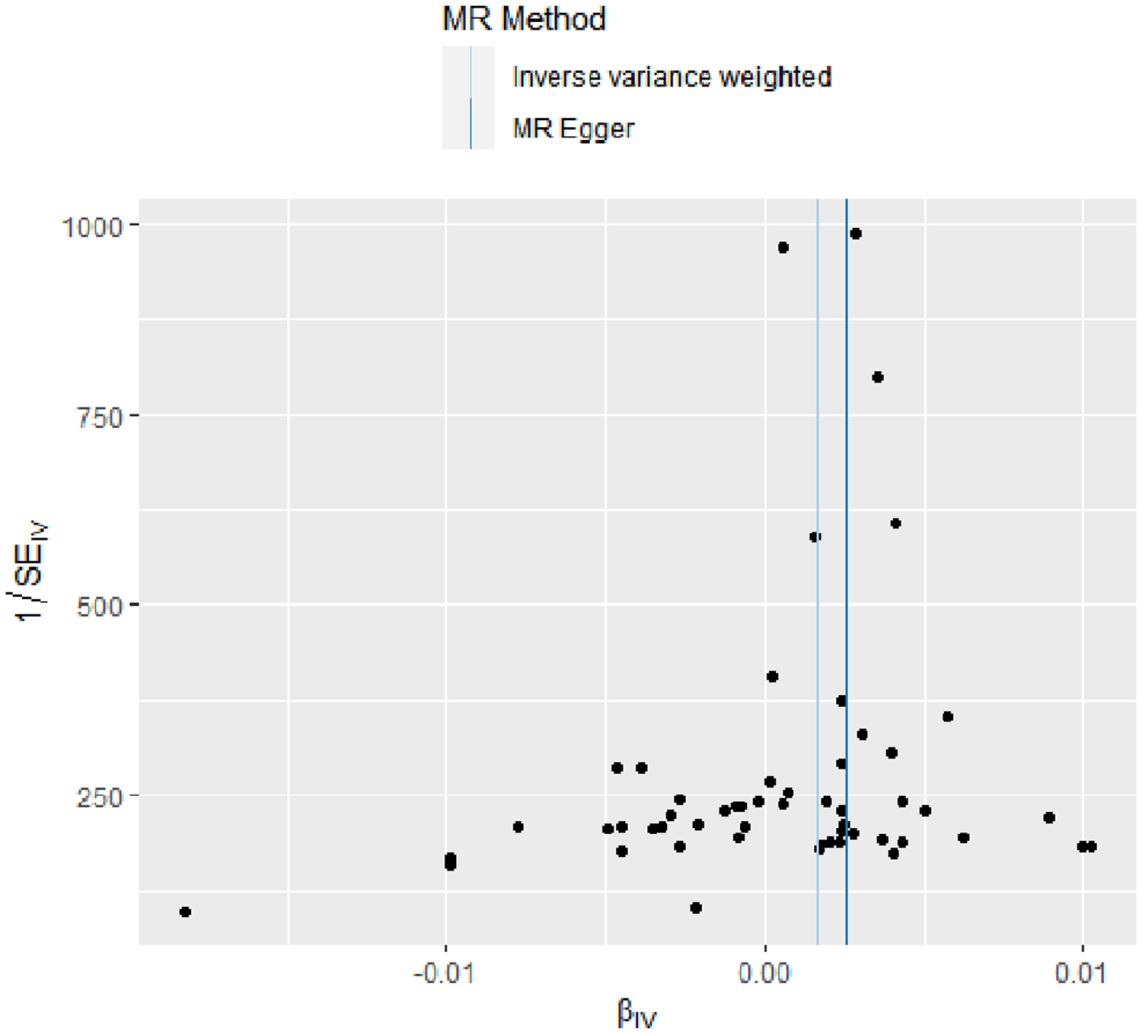
Funnel plot for RA on coronary atherosclerosis. This plot can be used for visual inspection of symmetry, where any deviation can be suggestive of pleiotropy. Each SNP's MR estimate is plotted against its minor allele frequency corrected association with RA. We note that our plot appears generally symmetrical. SNPs, single nucleotide polymorphisms. MR, Mendelian randomization; RA, rheumatoid arthritis.

**Figure 5:**
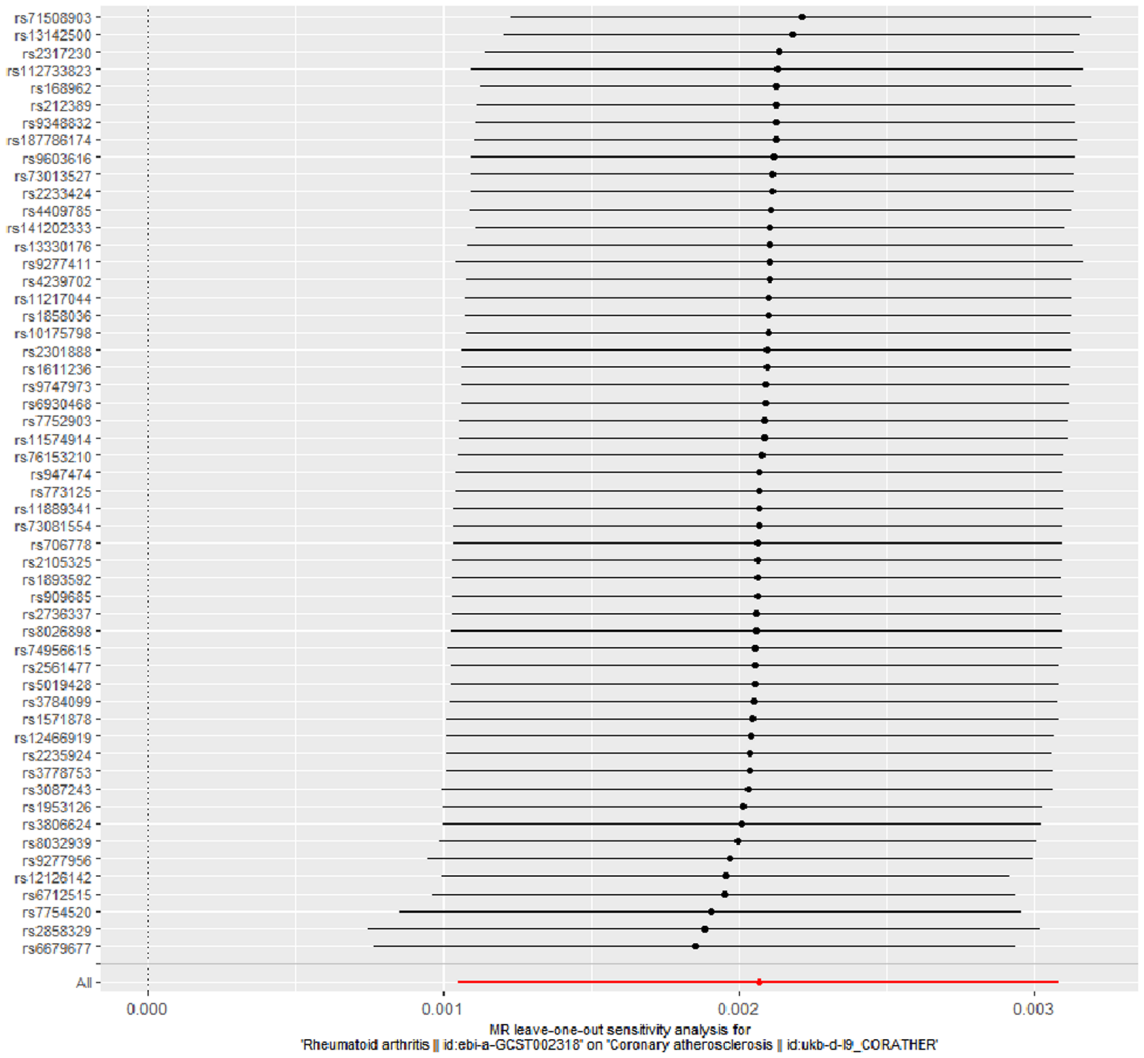
“Leave-one-out” analysis for RA on coronary atherosclerosis. “Leave-one-out” analysis of the causal effect of RA on coronary atherosclerosis susceptibility. Each black point represents the IVW effect estimate for the SNPs remaining after excluding individual SNP in turn, and the black line represents the corresponding 95% CI for the IVW effect estimate. The red point represents the IVW estimate using all SNPs. RA, rheumatoid arthritis; IVW, inverse variance weighted; SNPs, single nucleotide polymorphisms; CI, confidence interval.

The evidence of horizontal pleiotropy and heterogeneity was not obtained in this analysis. To be more specific, MR-Egger intercept (*P* = 0.20) and MR-PRESSO (*P* = 0.06) were not statistically significant, indicating the result did not bias by genetic pleiotropy (shown in Figure 3). In addition, the funnel plot showed that the causal effect of each SNP was symmetrically distributed around the IVW estimates, revealing that causal association was less likely to be affected by potential bias (shown in Figure 4). Cochran's Q test failed to discover significant heterogeneity between IVs estimates (*P* = 0.05). Furthermore, “Leave-one-out” analysis showed that no single SNP affected the overall estimation of causal association, validating the reliability of the findings of this study (shown in Figure 5).

### 3.4. Reverse MR

In this study, we also conducted a reverse MR study to find out whether there was a correlation between coronary atherosclerosis and RA. The result indicated genetic liability to atherosclerosis had no potential causal effect on RA (OR: 0.1384, 95%CI 0.0177–1.0823, *P* = 0.06). MR-Egger regression and weighted median and maximum likelihood ratio did not show a causal relationship between them (All *P* > 0.05).

### 3.5. Multivariate MR

Studies have shown that interleukin 1 (IL-1), interleukin 6 (IL-6), interleukin 8, interferon-*β* and tumor necrosis factor-alpha (TNF-α) are closely related to RA and coronary arteries (20). Therefore, we included these factors in the study to conduct multivariate MR. The results showed that RA was positively correlated with coronary atherosclerosis (OR: 1.0015, 95%CI 1.0004–1.0026, *P* < 0.05), and IL-6 was negatively correlated with coronary atherosclerosis (OR: 0.9911, 95%CI 0.9855–0.9967, *P* < 0.05), but the number of SNPS included was less. However, genetic liability to IL-1, interleukin 8, interferon-*β* and TNF-α had no potential causal effect on coronary atherosclerosis (All *P* > 0.05).

## 4. Discussion

In the present study, we discussed the causal association between RA and susceptibility to coronary atherosclerosis using MR analysis. Based on the GWAS pooled data from RA and coronary atherosclerosis, our results demonstrated that RA was positively related to the risk of coronary atherosclerosis.

Accumulating evidence indicates RA is associated with premature coronary atherosclerosis. As suggested by a case-control study, RA patients were more likely to have vulnerable non-calcified coronary plaque than controls (5). Other studies supporting the conclusion demonstrated that coronary plaque burden was higher and more severe in RA patients (49.7% vs. 45.8%, *P* < 0.05) (21). Besides, subclinical atherosclerosis was observed in patients with early RA. For instance, a prospective study showed a high annual standardized progression rate of carotid intima-media thickness in RA patients during 18 to 36 months of follow-up (*P* < 0.05) (22). Researchers observed a 3-fold increased risk of carotid plaques in RA patients in a case-sectional study of 98 outpatients with connective tissue diseases and 98 matched controls (44% vs. 15%, *P* < 0.05) (23). However, some observational studies have drawn different conclusions. Fazeli Y et al. found that the pathogenesis of the atherosclerosis process preceded the diagnosis of RA (24). Another study demonstrated that coronary artery calcification was no higher in RA patients compared to non-RA patients (64% vs. 52%, *P* = 0.31) (25). Therefore, due to environmental and genetic influences, the causality relationship between RA and coronary atherosclerosis remains unclear in observational studies. In the MR analysis, the findings provided genetic evidence that RA may increase the risk of susceptibility to coronary atherosclerosis.

However, the cause of coronary atherosclerosis has not been fully elucidated in RA. The risk failed to be interpreted entirely by traditional cardiovascular risk factors (26). A prospective cohort study suggested that higher inflammation was an independent predictor of coronary plaque progression in RA patients (27). This was consistent with the previous study (28). Accumulated inflammation may promote plaque formation and remodeling, which is closely related to the extent and duration of inflammation (29). Therefore, as a non-traditional cardiovascular factor, inflammation is generally considered to be the primary cause of coronary atherosclerosis in RA patients.

There is evidence that RA patients have increased levels of pro-inflammatory cytokines, including TNF-α, IL-1, and IL-6 (30). To date, these cytokines are believed to play a vital role in developing and progressing atherosclerotic plaques. Specifically, IL-1 upregulates the expression of matrix metalloproteinases at the location of atherosclerotic plaque formation, leading to plaque instability (31). Furthermore, TNF-α causes endothelial dysfunction, which may contribute to the formation and rupture of atherosclerotic plaques (32). Meanwhile, TNF-α can affect inflammatory responses by regulating the expression of IL-1 and IL-6 and adhesion molecules (33). Additionally, these inflammatory factors may promote the formation of atherosclerotic plaques by reducing Nitric oxide production and tissue utilization (34). Therefore, inflammation may play a central role in the process of coronary atherosclerosis in RA patients.

Besides, disease-modifying antirheumatic drugs may improve cardiovascular outcomes by effectively inhibiting inflammation. For instance, novel anti-inflammatory biologics, such as TNF inhibitors and IL-1 inhibitors have also shown positive effects on cardiovascular outcomes. TNF inhibitors were found to reduce all-cause cardiovascular events in RA patients, which may be attributed to their direct effect on inflammation, according to a meta-analysis of 28 studies (35). Moreover, TNF inhibitors were shown to improve endothelial function independent of the inflammatory responses (36). Another larger, randomized, phase III trial demonstrated that recurrence of all-cause cardiovascular events was reduced in patients with prior myocardial infarction receiving IL-1 inhibitors, accompanied by a marked decrease in the level of high-sensitivity C-reactive protein (HR: 0.8, 95%CI 0.73–0.95; *P* = 0.05) (37). Moreover, prolonged disease-modifying antirheumatic drug treatment may have atheroprotective effects on RA (38). In short, a reduction in overall cardiovascular disease risk always implies an improvement in coronary status. This also illustrated the pivotal role of inflammation in the pathogenesis of coronary atherosclerosis in RA patients.

Immune dysregulation may also be associated with accelerated coronary atherosclerosis. Antibodies to citrullinated protein antigens and rheumatoid factor have been used to diagnose RA and serve as accurate markers of disease severity. Serum antibodies to citrullinated protein antigens are associated with increased cardiovascular mortality in RA patients (HR: 1.52, 95%CI 1.04–2.21) (39). The following mechanisms may be involved: Firstly, citrullination of the protein alters the clearance function of macrophages (40). Secondly, antibodies to citrullinated protein antigens is associated with pro-thrombosis and pro-oxidative atherosclerotic immune cell profiles (41). Rheumatoid factor positive has also been shown to be a significant predictor of morbidity and mortality of cardiovascular diseases (42). Additionally, immunity dysregulation can stimulate the production of IL-6 and IL-8 in synovial tissue. These findings also provide evidence that immune dysregulation may play a role in accelerated coronary atherosclerosis.

This study used a two-sample MR method to explore the causal relationship between RA and coronary atherosclerosis. Studies have suggested that RA may increase the susceptibility to coronary atherosclerosis. Compared with observational studies, our study showed some advantages: Firstly, our study minimizes unmeasured confusion and reverse causality bias. Secondly, we performed the IVW method MR-Egger regression, weighted median and maximum likelihood for the two-sample MR analysis, increasing the reliability of the conclusion. In addition, the multivariate MR analysis result was consistent with the two-sample MR analysis. Thirdly, the present study did not reveal potential horizontal pleiotropy, confirming the robustness of our findings. However, there were also some limitations to our analysis: Firstly, our study was limited to populations of Europe descent. Although we reduced the bias introduced by population stratification, it may be inapplicable in the population of other ethnicities. Therefore, the conclusion needs to be validated in clinical studies for different populations. Secondly, as with all MR studies, the presence of unobserved horizontal pleiotropy may affect the stability of the finding. Thirdly, the relatively small OR suggests that not only RA may affect coronary atherosclerosis, but also multiple factors or other unknown factors. Therefore, careful interpretation is necessary when extrapolating the result.

## 5. Conclusion

In summary, our MR study provided strong genetic evidence indicating RA may increase the susceptibility to coronary atherosclerosis, suggesting active intervention for rheumatoid arthritis may reduce the incidence of coronary atherosclerosis.

## Data Availability

The datasets presented in this study can be found in online repositories. The names of the repository/repositories and accession number(s) can be found in the article/**Supplementary Material**.
