# Supplementary material for: Rheumatoid arthritis and coronary atherosclerosis: a two-sample Mendelian randomization study
Source: Front Cardiovasc Med. 2023 Apr 28;10:1033644. doi: 10.3389/fcvm.2023.1033644 (PMC10175685; doi:10.3389/fcvm.2023.1033644)
Supplement: Supplementary file 2 [file Table2.docx]

**Table S2.** Characteristics of SNPs included in MR analysis.

| RA-Associated SNPs | Nearest Gene(s) | Chr | Allele | Allele  Frequency | F |
| --- | --- | --- | --- | --- | --- |
| rs10175798 | *LBH* | 2 | A | 0.6203 | 38.7020 |
| rs11217044 | *RP11-158I9.1* | 11 | C | 0.2028 | 51.6969 |
| rs112733823 | *LINC00243* | 6 | T | 0.8718 | 229.3957 |
| rs11574914 | *CCL21* | 9 | A | 0.6849 | 40.0679 |
| rs11889341 | *STAT4* | 2 | T | 0.7714 | 55.4561 |
| rs12126142 | *IL6R* | 1 | A | 0.3598 | 25.9393 |
| rs12466919 | *LINC01185* | 2 | T | 0.6382 | 70.6139 |
| rs13142500 | *TAPT1-AS1* | 4 | C | 0.4930 | 30.4058 |
| rs13330176 | *RP11-542M13.2* | 16 | A | 0.2396 | 70.6139 |
| rs141202333 | *NRM* | NA | NA | NA | 33.4051 |
| rs1571878 | *CCR6* | 6 | C | 0.4215 | 165.3450 |
| rs1611236 | *NA* | NA | NA | NA | 105.6354 |
| rs168962 | *ZFP36L1* | 4 | C | 0.7664 | 25.9393 |
| rs1858036 | *SPRED2* | 2 | A | 0.6610 | 66.9308 |
| rs187786174 | *MMEL1* | 1 | A | 0.3121 | 47.4670 |
| rs1893592 | *UBASH3A* | 21 | A | 0.7237 | 55.7327 |
| rs1953126 | *PHF19* | 9 | C | 0.6402 | 36.6285 |
| rs2105325 | *RP11-296O14.2* | 1 | A | 0.2455 | 37.1051 |
| rs212389 | *ZNF831* | 20 | G | 0.3738 | 27.3536 |
| rs2233424 | *TMEM151B* | 6 | T | 0.0497 | 70.1923 |
| rs2235924 | *PADI2* | 1 | G | 0.6869 | 32.4788 |
| rs2301888 | *PADI4* | 1 | G | 0.6392 | 55.8626 |
| rs2317230 | *FCRL3* | 1 | G | 0.5378 | 30.3098 |
| rs2561477 | *C5orf30* | 5 | A | 0.3022 | 57.7459 |
| rs2736337 | *BLK* | 8 | C | 0.2445 | 84.4420 |
| rs2858329 | *TBC1D22B* | 6 | G | 0.5139 | 759.0836 |
| rs3087243 | *CTLA4* | 2 | A | 0.4702 | 64.8244 |
| rs3778753 | *IRF5* | 7 | G | 0.4751 | 37.1051 |
| rs3784099 | *RAD51B* | 14 | A | 0.3439 | 32.4788 |
| rs3806624 | *EOMES* | 3 | G | 0.4672 | 55.2895 |
| rs4239702 | *CD40* | 20 | C | 0.7127 | 100.9924 |
| rs4409785 | *RP11-338H14.1* | 11 | C | 0.1899 | 37.1051 |
| rs5019428 | *PLCL2* | 3 | A | 0.5229 | 38.7020 |
| rs6679677 | *PHTF1* | 1 | A | 0.0915 | 722.9848 |
| rs6712515 | *LINC01104* | 2 | C | 0.5408 | 55.7327 |
| rs6930468 | *RP11-157J24.2* | 6 | A | 0.3499 | 69.1872 |
| rs706778 | *IL2RA* | 10 | T | 0.4135 | 38.7020 |
| rs71508903 | *ARID5B* | 10 | C | 0.7803 | 83.7968 |
| rs73013527 | *RP11-744N12.3* | 11 | C | 0.4702 | 32.4788 |
| rs73081554 | *RP11-80H18.3* | 3 | C | 0.9483 | 31.6882 |
| rs74956615 | *RAVER1* | 19 | A | 0.0298 | 56.6519 |
| rs76153210 | *AARS2* | 6 | C | 0.9841 | 48.0415 |
| rs773125 | *SUOX* | 12 | A | 0.6113 | 36.6285 |
| rs7752903 | *AL356739.1* | 6 | G | 0.0199 | 126.8913 |
| rs7754520 | *XXbac-BPG116M5.17* | 6 | T | 0.0895 | 378.9503 |
| rs8026898 | *PCAT29* | 15 | A | 0.2853 | 129.8024 |
| rs8032939 | *RASGRP1* | 15 | C | 0.2416 | 44.3718 |
| rs909685 | *SYNGR1* | 22 | T | 0.3012 | 40.0679 |
| rs9277411 | *HLA-DPB1* | 6 | C | 0.6869 | 459.0442 |
| rs9277956 | *HTATSF1P1* | 6 | C | 0.1531 | 130.9406 |
| rs9348832 | *MAS1L* | 6 | A | 0.0278 | 68.8073 |
| rs947474 | *RP11-563J2.3* | 10 | A | 0.8211 | 33.3831 |
| rs9603616 | *COG6* | 13 | C | 0.6620 | 39.6638 |
| rs9747973 | *GRB7* | 7 | C | 0.5179 | 72.2966 |
| rs72831345 | *CABCOCO1* | 10 | A | 0.1292 | 38.7020 |
| rs9330353 | *PCDH18* | 4 | A | 0.4145 | 51.6969 |
| rs1275985 | *KCNK* | 2 | C | 0.4066 | 229.3957 |
| rs12258967 | *CACNB2* | 10 | C | 0.7137 | 40.0679 |
| rs17035646 | *CASZ1* | 1 | A | 0.3529 | 55.4561 |
| rs6991641 | *CTD-2135J3.4* | 8 | C | 0.6004 | 25.9393 |

RA, rheumatoid arthritis; SNP, single nucleotide polymorphisms.
